# Effects of dance-based physical activity on cognitive and psychological health across age groups: a systematic review

**DOI:** 10.3389/fpsyg.2026.1838599

**Published:** 2026-10-05

**Authors:** Yingying Fan, Xutao Liu

**Affiliations:** School of Physical Education, Jiangsu University of Science and Technology, Zhenjiang, Jiangsu, China

**Keywords:** cognitive function, dance-based physical activity, lifespan, mental health, systematic review

## Abstract

**Background:**

Dance-based physical activity is a multimodal intervention integrating motor, cognitive, emotional, and social components, and has been increasingly recognized for its potential to enhance cognitive function and mental health. However, evidence regarding its effectiveness across different age groups remains fragmented. This systematic review aimed to synthesize current evidence on the effects of dance-based interventions on cognitive and psychological health across different age groups.

**Methods:**

This systematic review was conducted in accordance with PRISMA 2020 guidelines and registered in PROSPERO (CRD420261338912). Six electronic databases (PubMed, Web of Science Core Collection, Scopus, APA PsycINFO, Cochrane Central Register of Controlled Trials (CENTRAL), and SPORTDiscus) were systematically searched from database inception to 28 August 2026. Eligible studies included randomized controlled trials (RCTs) and controlled clinical trials (CCTs) that examined the effects of dance-based interventions on cognitive and/or psychological outcomes in participants of any age. Due to substantial heterogeneity in intervention types, outcome measures, and study designs, a narrative synthesis was performed.

**Results:**

A total of 22 studies involving 1,645 participants aged 4 to 94 years were included. Favorable findings were reported for selected cognitive outcomes, including global cognition, memory, and attention, whereas findings for executive function were mixed. Some studies also reported favorable changes in depressive symptoms, anxiety, stress, emotional well-being, subjective well-being, and quality of life. However, these findings were not consistent across all studies or outcome measures, and differences in outcome measures and statistical reporting prevented reliable comparison of effect magnitude across studies. Several favorable findings were derived from studies judged to have a high, serious, or critical risk of bias. The evidence was predominantly derived from older adults, particularly those with cognitive impairment, whereas evidence involving children, adolescents, and younger or middle-aged adults remained limited and heterogeneous; therefore, definitive comparisons across age groups could not be made.

**Conclusion:**

Current evidence suggests that dance-based physical activity may have favorable effects on selected cognitive and psychological outcomes, with the evidence most extensively derived from older adults, particularly those with cognitive impairment. However, methodological heterogeneity, small sample sizes, study-level risk of bias, and limited long-term follow-up restrict the strength of these conclusions. Evidence involving children, adolescents, and younger or middle-aged adults remains insufficient for meaningful comparisons across age groups. Further high-quality trials are needed to determine optimal intervention parameters and clarify the mechanisms underlying the potential cognitive and psychological effects of dance-based physical activity.

**Systematic review registration:**

https://www.crd.york.ac.uk/prospero/display_record.php?ID=CRD420261338912, CRD420261338912.

## 1 Introduction

Cognitive decline and mental health problems are major public health challenges in contemporary society. With the accelerating pace of global population ageing, the prevalence of mild cognitive impairment (MCI) among older adults continues to rise, posing serious threats to their quality of life and social functioning (World Health Organization, 2021). Progressive cognitive impairment may also increase the likelihood of psychological problems in affected individuals, thereby further undermining both physical and mental well-being. Although pharmacological treatments may be beneficial for some conditions, their effectiveness and applicability can be limited by adverse effects, treatment burden, and costs. Therefore, there is continued interest in safe and accessible non-pharmacological approaches.

Physical activity is a relatively low-cost and accessible non-pharmacological approach that has shown potential for preventing or delaying cognitive decline. The WHO Guidelines for Risk Reduction of Cognitive Decline and Dementia published in 2019 recommend physical activity for cognitively healthy adults to reduce the risk of cognitive decline, and likewise recommend physical activity for individuals with mild cognitive impairment (MCI) for the same purpose (World Health Organization, 2019). Compared with conventional exercise, dance provides a distinctive combination of physical activity, cognitive engagement, rhythmic stimulation, and social interaction (Fong Yan et al., 2018). In addition, with advancing age, cognitive functions such as attention, memory, and concentration gradually deteriorate, becoming slower and less efficient, while physical functions such as walking and balance show similar age-related decline (Sofi et al., 2011).

Dance places relatively high demands on individuals’ motor planning, motor memory, multi-task coordination and processing, as well as their capacity for sustained and conscious attentional control (Kalyani et al., 2019). As a distinctive form of physical activity, dance differs from conventional aerobic exercise and resistance training (Jaldin et al., 2024) in that it integrates musical rhythm, social interaction, and cognitive engagement. It also requires individuals to remember the sequence of dance movements (Wu et al., 2021), thereby providing a unique intervention model for improving cognitive function and psychological well-being.

Dance-based physical activity is being increasingly applied in the fields of rehabilitation and geriatrics. Evidence suggests that dance-based interventions have shown varying degrees of therapeutic benefit in diverse clinical populations, including individuals with Parkinson’s disease, cognitive decline, depression, and specific learning disorders, generally demonstrating promising treatment trends. Particularly among older adults, dance interventions can not only improve physical function (Ambegaonkar et al., 2022) and balance ability (Wang et al., 2023; Wu and Zhang, 2025), thereby reducing the risk of injury and falls, but also enhance social participation and interpersonal relationships through the promotion of positive emotional expression (Ambegaonkar et al., 2022; Cofini et al., 2021), and may contribute to improvements in depressive symptoms and emotional well-being. Intervention duration may also influence outcomes, although the optimal duration and overall dosage of dance-based interventions remain unclear.

Previous systematic reviews have primarily examined the effects of dance interventions in specific populations, particularly older adults and individuals with mild cognitive impairment or dementia (Jaldin et al., 2024; Wu et al., 2021; Prates et al., 2025; Tao et al., 2023). Other reviews have focused on specific outcomes, such as cognitive function, depression, anxiety, physical function, or quality of life (Hewston et al., 2020; Prudente et al., 2024; Lu et al., 2024). Although some reviews have also explored the physical and psychological benefits of dance in children and adolescents (Tao et al., 2022), the available evidence remains largely organized around specific populations, age groups, or health outcomes. Consequently, a comprehensive synthesis of both cognitive and psychological outcomes across different age groups remains limited.

Therefore, this systematic review aimed to synthesize the available evidence on the effects of dance-based physical activity on cognitive function and psychological health in children, adolescents, young adults, middle-aged adults, and older adults. By considering both outcome domains and examining the distribution of evidence across age groups, this review sought to identify potential benefits, methodological limitations, and underrepresented populations. Given the uneven availability of studies across age groups, age-related findings were interpreted descriptively rather than as definitive comparisons of intervention effectiveness.

## 2 Materials and methods

### 2.1 Protocol and registration

This review followed the PRISMA 2020 guidelines (Page et al., 2020) and was registered in PROSPERO, with the registration number CRD420261338912. The risk of bias of included RCTs was assessed using the Cochrane RoB 2 tool (Sterne et al., 2019). The risk of bias of the included non-randomized controlled studies was assessed using the Risk Of Bias In Non-randomized Studies of Interventions (ROBINS-I) tool (Sterne et al., 2016).

### 2.2 Search strategy

The literature search was conducted across six international databases: Web of Science Core Collection, PubMed, Cochrane CENTRAL, SPORTDiscus with Full Text, APA PsycINFO, and Scopus. APA PsycINFO and SPORTDiscus with Full Text were searched via EBSCOhost. These databases were selected because they cover relevant fields, including medicine, psychology, rehabilitation, sports science, and multidisciplinary research, thereby reducing the risk of missing relevant studies. The databases were searched from inception to 28 August 2026, with no restrictions on participant age. The search keywords used were as follows: (“dance” OR “dance therapy” OR “dance movement therapy” OR “dance-based exercise” OR “dance intervention” OR “dance-based physical activity”) AND (“cognition” OR “cognitive function” OR “executive function” OR “memory” OR “attention” OR “psychological health” OR “mental health” OR “depression” OR “anxiety” OR “well-being” OR “self-esteem” OR “quality of life”) AND (“random” OR “controlled trial” OR “control group”). To retain sensitivity for CCTs, the study-design block included “controlled trial” and “control group” in addition to “random,” rather than being restricted to randomized designs. Eligible CCTs were required to include a concurrent comparator and were therefore expected to be identifiable through these control-related terms in the searched fields. We also verified that all four CCTs ultimately included in the review contained at least one of these terms and were retrieved by the revised searches. Although no study-design filter can completely exclude the possibility of missing poorly indexed records, the use of multiple control-related terms where the filter was applied was not expected to materially reduce sensitivity for CCT designs. No separate study-design block was applied in CENTRAL because it is a controlled-trials register.

Table 1 presents the search terms used in the databases, and Table 2 provides the complete search strategies for all databases.

**Table 1:** Conceptual search terms used across databases.

Dance-based intervention	Operator	Cognitive or psychological outcomes	Operator	Study design
(“dance” OR “dance therapy” OR “dance movement therapy” OR “dance-based exercise” OR “dance intervention” OR “dance-based physical activity”)	AND	(“cognition” OR “cognitive function” OR “executive function” OR “memory” OR “attention” OR “psychological health” OR “mental health” OR “depression” OR “anxiety” OR “well-being” OR “self-esteem” OR “quality of life”)	AND	(“random” OR “controlled trial” OR “control group”)

The study-design search block was applied where appropriate and was not required for CENTRAL.

**Table 2:** Database-specific search strategies and number of records retrieved.

Database	Complete search strategy	Results
Web of Science Core Collection (From database inception-2026.8.28)	TS = (dance OR dancing OR “dance therapy” OR “dance-based” OR Zumba OR tango OR salsa) AND TS = (cognition OR cognitive OR “executive function” OR memory OR attention OR depression OR depressive OR anxiety OR “mental health” OR “psychological well-being” OR wellbeing OR “quality of life” OR “self-esteem”) AND TS = (random OR “controlled trial” OR “control group”)	966
PubMed (From database inception-2026.8.28)	(“Dancing”[MeSH] OR “Dance Therapy”[MeSH] OR dance[Title/Abstract] OR Dancing[Title/Abstract] OR “Dance-based”[Title/Abstract] OR Zumba[Title/Abstract] OR tango[Title/Abstract] OR salsa[Title/Abstract]) AND (“Cognition”[MeSH] OR “Executive Function”[MeSH] OR “Memory”[MeSH] OR “Attention”[MeSH] OR “Mental Health”[MeSH] OR “Depression”[MeSH] OR “Anxiety”[MeSH] OR “Quality of Life”[MeSH] OR cognition[Title/Abstract] OR cognitive[Title/Abstract] OR “executive function”[Title/Abstract] OR memory[Title/Abstract] OR attention[Title/Abstract] OR depression[Title/Abstract] OR anxiety[Title/Abstract] OR “mental health”[Title/Abstract] OR “psychological well-being”[Title/Abstract] OR wellbeing[Title/Abstract] OR “quality of life”[Title/Abstract] OR “self-esteem”[Title/Abstract]) AND (“Randomized Controlled Trial”[Publication Type] OR “Controlled Clinical Trial”[Publication Type] OR random[Title/Abstract] OR “controlled trial”[Title/Abstract] OR “control group”[Title/Abstract]) NOT (animals[MeSH Terms] NOT humans[MeSH Terms])	818
Cochrane CENTRAL (From database inception-2026.8.28)	(Mesh descriptor: [Dancing] explode all trees OR Mesh descriptor: [Dance Therapy] explode all trees OR dance:ti,ab,kw OR Dancing:ti,ab,kw OR Zumba:ti,ab,kw OR tango:ti,ab,kw OR salsa:ti,ab,kw OR “dance-based”:ti,ab,kw OR “dance therapy”:ti,ab,kw) AND (Mesh descriptor: [Cognition] explode all trees OR MeSH descriptor: [Memory] explode all trees OR MeSH descriptor: [Attention] explode all trees OR MeSH descriptor: [Executive Function] explode all trees OR MeSH descriptor: [Mental Health] explode all trees OR MeSH descriptor: [Depression] explode all trees OR MeSH descriptor: [Anxiety] explode all trees OR MeSH descriptor: [Quality of Life] explode all trees OR cognition:ti,ab,kw OR memory:ti,ab,kw OR attention:ti,ab,kw OR “executive function”:ti,ab,kw OR “mental health”:ti,ab,kw OR depression:ti,ab,kw OR anxiety:ti,ab,kw OR “quality of life”:ti,ab,kw)	1,189
SPORTDiscus with Full Text (From database inception-2026.8.28)	(TI (dance OR “dance therapy” OR “dance-based” OR Zumba OR tango OR salsa) OR AB (dance OR “dance therapy” OR “dance-based” OR Zumba OR tango OR salsa)) AND (TI (cognition OR “executive function” OR memory OR attention OR depression OR anxiety OR “mental health” OR wellbeing OR “quality of life” OR “self-esteem”) OR AB (cognition OR “executive function” OR memory OR attention OR depression OR anxiety OR “mental health” OR wellbeing OR “quality of life” OR “self-esteem”)) AND (TI (random OR “controlled trial” OR “control group”) OR AB (random OR “controlled trial” OR “control group”))	166
APA PsycINFO (From database inception-2026.8.28)	(SU (dance OR dancing OR “dance therapy” OR “dance-based”) OR TI (dance OR “dance therapy” OR Zumba OR tango OR salsa) OR AB (dance OR dancing OR “dance therapy” OR “dance-based” OR Zumba OR tango OR salsa)) AND (SU (cognition OR memory OR attention OR depression OR anxiety OR “mental health” OR “quality of life” OR “self-esteem”) OR TI (cognition OR “executive function” OR memory OR attention OR depression OR anxiety OR “mental health” OR wellbeing OR “quality of life” OR “self-esteem”) OR AB (cognition OR “executive function” OR memory OR attention OR depression OR anxiety OR “mental health” OR wellbeing OR “quality of life” OR “self-esteem”)) AND (TI (random OR “controlled trial” OR “control group”) OR AB (random OR “controlled trial” OR “control group”))	353
Scopus (From database inception-2026.8.28)	TITLE-ABS-KEY ((“dance” W/3 (intervention OR interventions OR training OR program OR therapy OR exercise)) OR “dance-based” OR Zumba OR tango OR salsa) AND TITLE-ABS-KEY (cognition OR cognitive OR memory OR attention OR “executive function” OR depression OR depressive OR anxiety OR anxious OR “mental health” OR “quality of life”) AND TITLE-ABS-KEY (random OR “controlled trial” OR “control group”)	720

### 2.3 Eligibility criteria and study selection

The literature was identified using the PICOS framework, which includes: (1) population, (2) intervention, (3) comparison, (4) outcomes, and (5) study design. Each PICOS component served as a criterion for the inclusion of relevant studies. To be considered eligible, a study had to meet all of the inclusion criteria presented in Table 3. All records identified through the database searches were imported into EndNote, and duplicate records were removed. Titles and abstracts were initially screened by one reviewer against the predefined eligibility criteria, and all screening decisions were subsequently verified by a second reviewer. Full texts of potentially eligible reports were sought through the university library’s subscribed databases and Google Scholar. When full text was not directly accessible, Google Scholar searches were conducted using the exact article title together with the first author’s name and publication year. The DOIs of reports that remained inaccessible were additionally checked in Unpaywall to identify potential open-access versions. Reports that remained unavailable after these retrieval attempts were classified as not retrieved. Retrieved full texts were then assessed against the eligibility criteria, with disagreements resolved through discussion.

**Table 3:** Eligibility criteria according to the PICOS framework.

PICOS	Detailed inclusion criteria
Population	People of all ages, including healthy individuals and those with cognitive impairment (e.g., MCI, cognitive decline, or dementia), specific learning disabilities, or mental health problems.
Interventions	Any structured dance-based physical activity program, including dance exercise, dance therapy, dance movement therapy, ballroom dance, square dance, Latin dance, Zumba and culturally specific dance forms.
Comparisons	Usual care, wait-list, health education, no intervention, conventional exercise, or other active controls.
Outcomes	Eligible studies were required to report at least one cognitive or psychological outcome. Cognitive outcomes: Global cognitive function, memory, attention, and executive function. Psychological outcomes: Depressive symptoms, anxiety, emotional well-being, subjective well-being, and self-esteem. Other reported outcomes, including Quality of life, social participation, and physical function, were considered only when reported alongside an eligible cognitive or psychological outcome.
Study designs	RCT or CCT

### 2.4 Data extraction and quality assessment

After completion of the literature search, data were extracted from all eligible studies, including: (1) study characteristics (authors, title, year of publication, and study design); (2) sample size and control group; (3) participant characteristics (e.g., age and sex); (4) intervention characteristics (type, duration, frequency, and intensity); (5) study outcomes. These data were extracted into a standardized form by one author and subsequently checked by a second author. Any discrepancies were resolved through discussion.

Using the Cochrane RoB 2 tool (Sterne et al., 2019), the risk of bias was assessed for the 18 included randomized controlled trials. The assessment was based on five domains: (1) the randomization process; (2) deviations from intended interventions; (3) missing outcome data; (4) measurement of the outcome; (5) selection of the reported result. Each study was categorized as “low risk of bias,” “some concerns,” or “high risk of bias.” The overall risk of bias was determined based on a comprehensive judgment across all domains. The four included non-randomized controlled studies were assessed using the ROBINS-I tool (Sterne et al., 2016). The assessment covered seven domains: (1) bias due to confounding; (2) bias in the selection of participants into the study; (3) bias in the classification of interventions; (4) bias due to deviations from intended interventions; (5) bias due to missing data; (6) bias in the measurement of outcomes; and (7) bias in the selection of the reported result. Each domain was judged as having low risk, moderate risk, serious risk, or critical risk.

### 2.5 Data synthesis

Before deciding against pooling, we examined whether studies could be combined within the main cognitive and psychological outcome domains. Quantitative synthesis was not feasible even within these domains because global cognition was assessed using different instruments and under substantially different population, intervention, and comparator conditions; memory, attention, and executive-function outcomes were based on non-equivalent tests and subscales; and psychological outcomes were distributed across heterogeneous measures of depression, anxiety, stress, mood, self-related functioning, and well-being. For several individual outcomes, too few sufficiently comparable studies were available, and some studies did not report the comparative estimates or variance data required for pooling.

Owing to substantial methodological heterogeneity in participant characteristics, intervention types, comparator conditions, intervention duration, and outcome measures, a meta-analysis was not considered appropriate. A structured narrative synthesis was therefore conducted. The included studies were first grouped according to cognitive and psychological outcome domains and were subsequently examined across age groups. Cognitive outcomes were organized into global cognitive function, memory, executive function, and attention, while psychological outcomes were organized into depressive symptoms, emotional well-being, subjective well-being, and related psychosocial outcomes. Intervention characteristics, comparator conditions, study design, and risk of bias were considered when interpreting the findings. Where available, between-group findings and study-reported effect estimates were given priority over within-group changes. Findings were described as favorable, mixed, or non-significant, rather than drawing conclusions solely from the number of statistically significant studies. Because the evidence remained unevenly distributed across age groups, with most studies involving older adults, findings across age groups were compared descriptively and were not interpreted as definitive evidence of age-related differences. Age categories were not treated as prespecified numerical subgroups. Studies were described according to the population labels and age ranges reported in the original articles, and samples spanning conventional age boundaries were not reassigned to mutually exclusive categories. Accordingly, the age-related synthesis describes the distribution of evidence across reported study populations rather than formal comparisons of intervention effects between age groups.

## 3 Results

### 3.1 Study selection

Figure 1 illustrates the workflow of the record screening process. A total of 4,212 records were identified through database searching, including 818 from PubMed, 720 from Scopus, 353 from APA PsycINFO, 1,189 from Cochrane CENTRAL, 166 from SPORTDiscus, and 966 from Web of Science Core Collection. After removing 2,267 duplicate records, 1,945 records remained for further screening. Of these, 1,733 records were excluded during title and abstract screening, and 212 reports were sought for retrieval. Fifty-nine reports remained unavailable after these retrieval attempts. Their DOI records were additionally checked in Unpaywall; all were classified as closed, with no open-access full-text location identified. These reports were therefore classified as not retrieved, leaving 153 reports for full-text eligibility assessment. Following full-text assessment, 131 reports were excluded. Ultimately, 22 studies were included in the review. During title and abstract screening, records were excluded if they clearly did not meet the predefined eligibility criteria, including studies that did not involve a dance-based intervention, used an ineligible study design, did not assess cognitive or psychological outcomes, were of an ineligible publication type, or were not published in English. Records for which eligibility could not be determined from the title and abstract were retained for full-text assessment.

**Figure 1:**
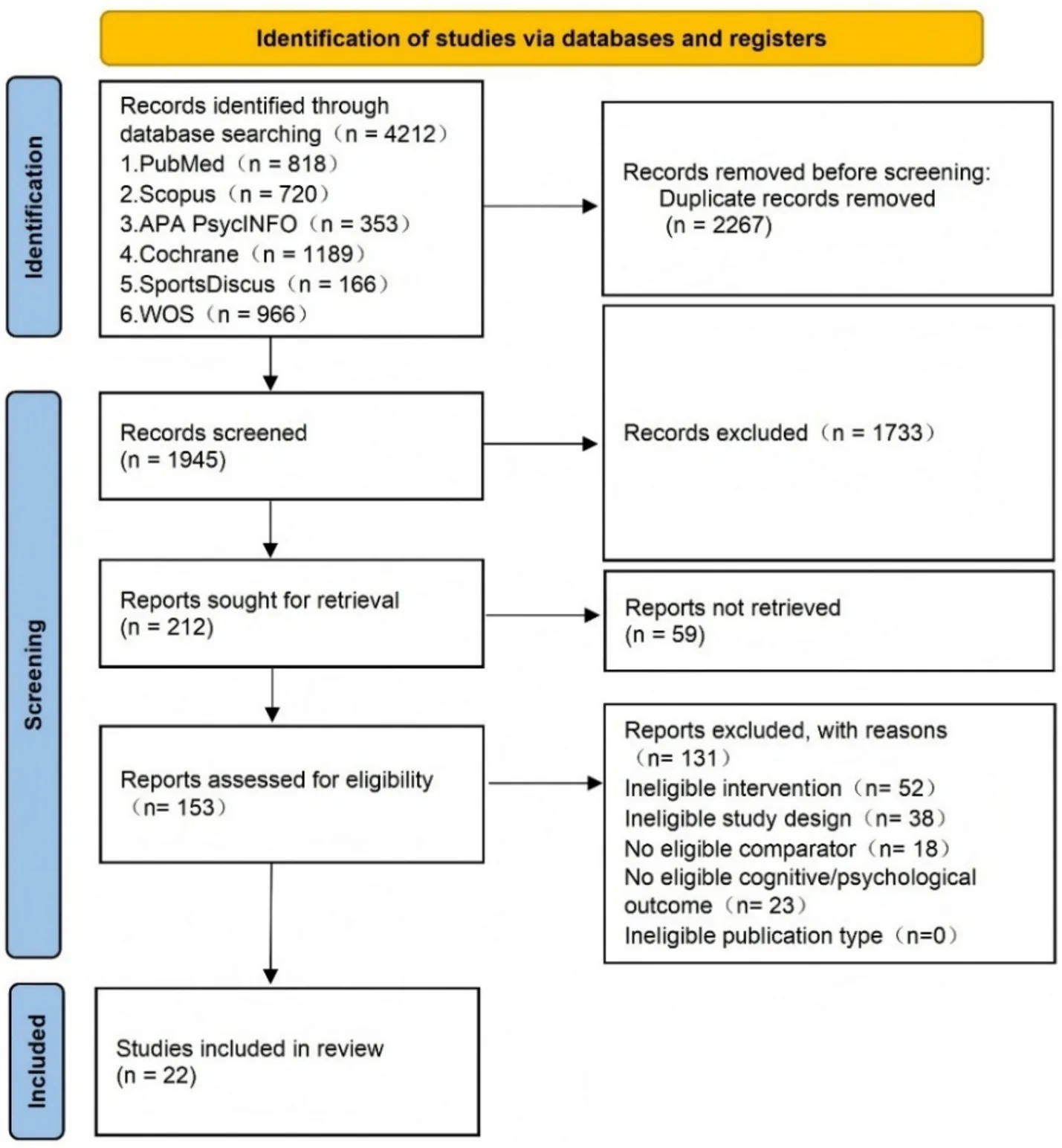
PRISMA flow diagram.

### 3.2 Risk of bias assessment

The risk of bias of the 18 included RCTs was assessed using RoB 2 (Figure 2). One study was rated as low risk of bias (Parial et al., 2023). Ten studies were rated as having some concerns, mainly related to the randomization process, deviations from intended interventions, and selection of the reported result (Ambegaonkar et al., 2022; Wu and Zhang, 2025; Cofini et al., 2021; Ayari et al., 2023; Sánchez-Alcalá et al., 2025; Sanprakhon et al., 2025; Tung et al., 2024; Ho et al., 2018; Luo et al., 2026; Dongquan et al., 2026). Seven studies were rated as high risk of bias, mainly because of serious concerns regarding deviations from intended interventions and missing outcome data (Wang et al., 2023; Kattenstroth et al., 2013; Hola et al., 2024; Zheng and Ji, 2021; Pinniger et al., 2012; Delextrat et al., 2016; Adey-Wakeling et al., 2026). For the four included non-randomized controlled studies, three studies (Hirahara et al., 2025; Zhao et al., 2021; Zach et al., 2015) were judged to have a serious overall risk of bias, whereas Aithal was judged to have a critical overall risk of bias (Aithal et al., 2019). These judgments were mainly driven by bias due to confounding and selection of participants, with missing data also contributing to the ratings in some studies. Detailed information on the risk-of-bias assessments is presented in Figures 2, 3. These risk-of-bias assessments were considered when interpreting the reported outcomes, particularly when favorable findings were derived from studies judged to be at high, serious, or critical risk of bias.

**Figure 2:**
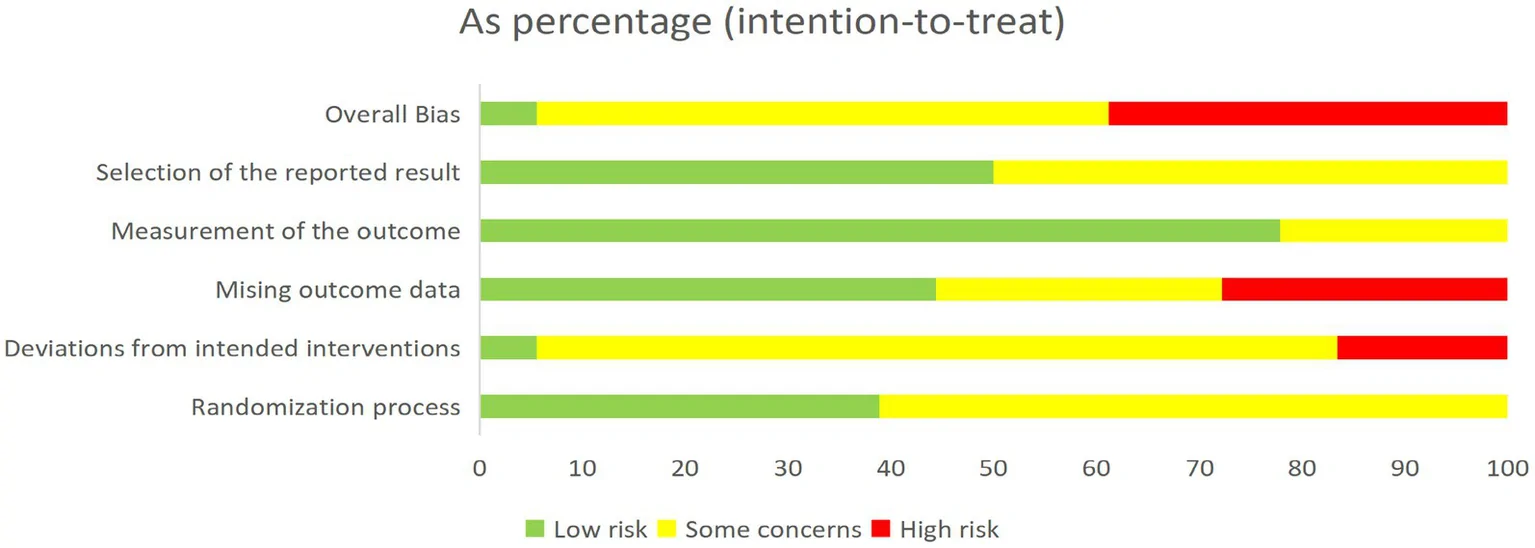
RoB 2 assessment of the included randomized controlled trials.

**Figure 3:**
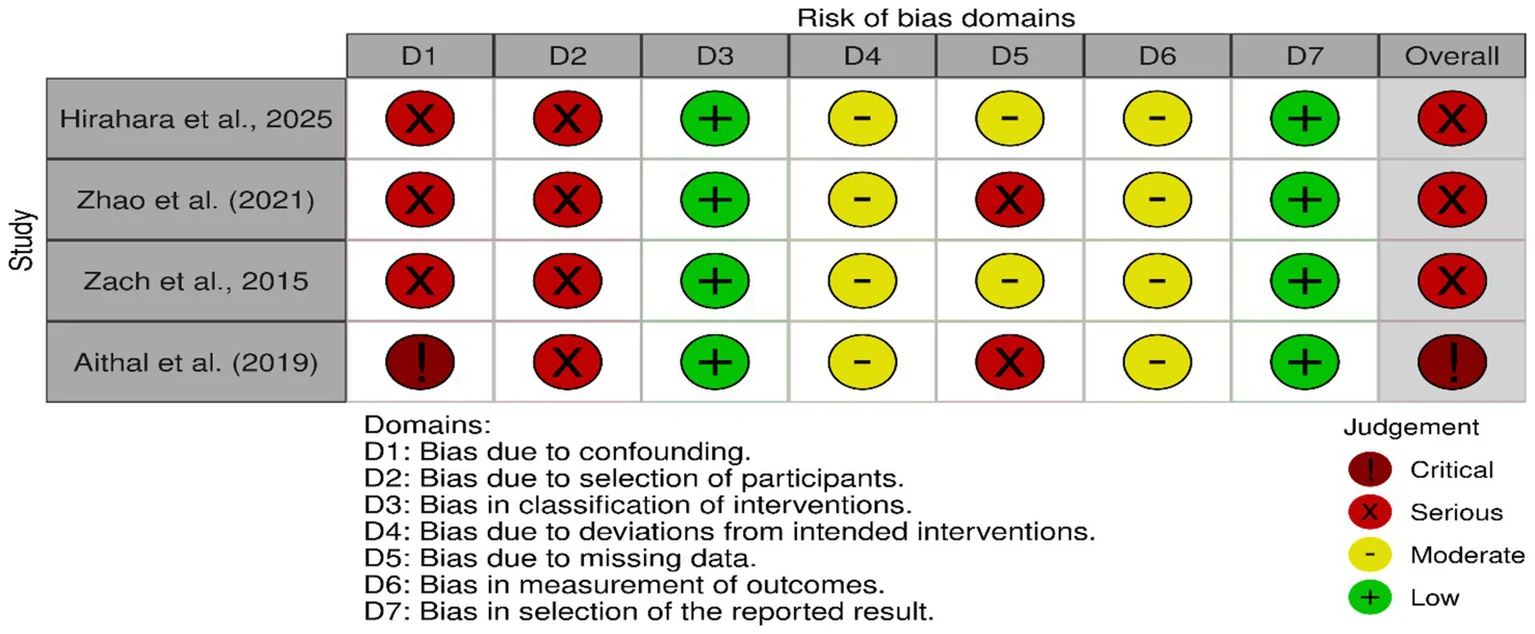
ROBINS-I risk of bias assessment for the included non-randomized controlled studies.

### 3.3 Participant characteristics

Across the 22 included studies, a total of 1,645 participants were involved. The age of the participants ranged from 4 to 94 years. The smallest study included 11 participants, whereas the largest study included 204 participants.

Most studies examined the effects of dance on cognitive function and emotional outcomes, such as depressive symptoms and subjective well-being. Thirteen studies involved older adults, three involved children, one involved adolescents, one involved college students, and four involved adult or middle-aged populations. The evidence base was therefore predominantly derived from older populations, with substantially less evidence available for children, adolescents, and younger or middle-aged adults. In addition, participants presented with diverse health conditions, including individuals with cognitive impairment, mild cognitive impairment, subjective cognitive decline, mild dementia, specific learning disabilities, and self-reported depression, whereas some studies involved healthy individuals (see Table 4).

**Table 4:** Characteristics and descriptive outcomes of the included studies.

Author (year)	Design	N	Population	Intervention						Outcome
				Age	Sex	Type	Frequency /Intensity	Duration	Comparator	
Ambegaonkar et al. (2022)	RCT	64	Older adults	71.3 ± 4.6	M: 26 F: 38	Ballroom dance	Twice per week for 1 h per session/NR	10 weeks	Social conversation and ukulele	Cognitive function ↑ over time in all groups Physical function ↑ Social engagement ↑ Between groups ↔
Ayari et al. (2023)	RCT	23	Seniors with cognitive impairment	78 ± 7	M: 7 F: 16	Dance	1 session per week, each session lasted 60 min/60–70% HRR	16 weeks	Aerobic exercise	Cognitive function ↑ (MMSE and recall) Depressive symptoms↓ (over time in both groups)
Cofini et al. (2021)	RCT	30	SLD children	9–11	M: 27 F: 3	Dance therapy+Traditional cognitive treatments	Once per week for 60 min per session/NR	6 months	Traditional cognitive treatments	Cognitive function ↑ Social engagement ↑ (interpersonal relationships) Attention, concentration and self-esteem ↑ Emotion recognition ↑ Other dimensions ↔
Ho et al. (2018)	RCT	204	Older adults	79 ± 8	M: 81.9%	Dance movement therapy	1-h sessions for twice a week/40–60% of the VO2 max value	12 weeks	Physical exercise and Waitlist control group	Psychosocial function ↑ Depression, loneliness, and negative mood↓ versus waitlist at post-intervention Cognitive function ↔
Hola et al. (2024)	RCT	77	Older adults	70.3 ± 3.8	F: 83.1%	Dance	Twice a week, lasted 90 min per session/NR	12 weeks	Martial arts and inactive usual-lifestyle control	Cognitive function ↑ Attention↑ Depressive symptoms↓versus inactive control
Kattenstroth et al. (2013)	RCT	35	Older adults	60–94	M: 11 F: 24	Dance	1 h/week/NR	6 months	Maintain usual lifestyle	Cognitive function and Attention↑ within the dance group Subjective well-being ↑
Parial et al. (2023)	RCT	60	People with MCI	63.8 ± 5.24	F: 76.7%	Dual-task Zumba Gold	Thrice weekly for 60 min/Moderate intensity	12 weeks	Health education	Cognitive function, executive function, immediate/delayed recall, and quality of life↑ Subjective well-being ↑ Depressive symptoms ↔
Sánchez-Alcalá et al. (2025)	RCT	92	Older adults with MCI	71.83 ± 2.96	M: 36.96% F: 63.04%	Dance	Two sessions per week for 60 min/Moderate intensity	12 weeks	Maintain usual lifestyle	Cognitive function ↑ Verbal Fluency ↑ Cognitive Impairment ↓
Sanprakhon et al. (2025)	RCT	108	Older adults with MCI and subjective cognitive decline	60–85	M: 16 F: 92	Traditional Thai folk-dance combined with cognitive stimulation	90 min per session/Moderate intensity	7 weeks	Routine care	Cognitive function ↑ Quality of life ↑ Mild behavioral impairment ↓ versus routine care
Tung et al. (2024)	RCT	60	Older adults	≥55	M: 13 F: 47	Digital somatosensory dance	Twice a week, each session ≥ 30 min/NR	6 months	Health education	Cognitive function (MoCA total) ↑ before but ↔ after FDR correction Quality of life ↑ Weight ↓
Wang et al. (2023)	RCT	34	Older adults	60–80	M: 8 F: 26	Dance	Three dance sessions weekly for 60 min/Moderate intensity	12 weeks	Maintain usual lifestyle	Balance ↑ Cognitive function ↑
Wu et al. (2025)	RCT	107	Older adults	69.9 ± 7.5	M: 39 F: 68	Square dance and Latin dance	Three times a week, 60 min per session/NR	12 weeks	Tai Chi, Baduanjin, and Health education	Balance ↑ within the square- and Latin-dance groups only Cognitive function ↑ (square dance) Psychological well-being ↑ Weight ↓within the Latin-dance group only
Zheng and Ji (2021)	RCT	181	College students	19.56 ± 1.30	M: 86 F: 95	Sports dance (Waltz + Cha-cha)	Twice per week, 90 min per session/Moderate intensity	8 weeks	No systematic exercise	Self-satisfaction ↑ Negative emotional reaction ↓; Interpersonal communication ↓ Psychological stress ↓ Learning disorder ↓
Luo et al. (2026)	RCT	86	Rural left-behind children	10.39 ± 0.87	M: 48 F: 38	Line dance only/ with visual stimulation/with multisensory stimulation	Three times per week, 60 min per session/Moderate (40–59% HRR)	12 weeks	Regular PE classes	Social anxiety ↓ (visual/multisensory groups vs. control)
Pinniger et al. (2012)	RCT	66	Adults with self-reported depression	44.39 ± 14.27	M: 6 F: 60	Argentine tango dance	1.5 h per week/NR	6 weeks	Mindfulness meditation and Waiting-list control	Mindfulness ↑ (tango vs. meditation/waitlist) Depression ↓ (tango and meditation vs. waitlist) Stress ↓ (tango vs. waitlist)
Delextrat et al. (2016)	RCT	37	Healthy women	27.23 ± 5.70	F: 37	Zumba	Three times per week, 60 min per session/HRmean 144 ± 11 bpm	8 weeks	No intervention	Physical strength self-perception ↑ Autonomy ↑ Purpose in life ↑ Other psychological well-being domains ↔
Dongquan et al. (2026)	RCT	111	Chinese adolescents	14.02 ± 0.81	M: 57 F: 63	Ballroom dance	3 sessions/week, 45 min/session/NR	12 weeks	Regular campus life	Anxiety symptoms ↓ Salivary cortisol ↓ (versus control at post-intervention and follow-up)
Adey-Wakeling et al. (2026)	RCT	43	Older adults with recent acquired brain injury	68.8 ± 13.4	NR for completers	Therapeutic adapted dance	Three times per week, 45 min/session/NR	10 weeks	Circuit physiotherapy	Emotional well-being ↑ (at follow-up ↔) Fatigue ↓ (at follow-up ↔) Anxiety/depression ↓ (at follow-up ↔) Self-efficacy ↔
Hirahara et al. (2025)	CCT	30	Middle-aged adults	30–64	M: 9 F: 21	Ballroom dance	60 min, once per week/3–5.5 METs	10 weeks	No dance lessons	Total mood disturbance ↓versus control Other mood subscales ↔
Zhao et al. (2021)	CCT	63	Older adults with MCI	72.29 ± 6.03	F: 82.54%	Square dancing	Three times per week for 60 min per session/60–80% of the maximum heart rate	3 months	Health education	Cognitive function ↑ Depressive symptoms↓ (effects were attenuated at follow-up)
Zach et al. (2015)	CCT	123	Kindergarten children	4–5 years	M: 60; F: 63	Dance	Weekly sessions, 9 sessions total/NR	9 weeks	Orienteering and no-intervention control	Attention ↑ Spatial perception ↑
Aithal et al. (2019)	CCT	11	Caregivers of children with ASD	28–35 years	F:11	Dance Movement Psychotherapy	90 min per session, 3 sessions per week/NR	2 weeks	Waiting-list control	Parenting Stress ↓ versus control Depression ↓ but the between-group effect was not established

HRR, Heart Rate Reserve; SLD, Specific Learning Disability; METs, Metabolic Equivalent of Task; MCI, Mild Cognitive Impairment; RCT, Randomized Controlled Trial; CCT, Controlled Clinical Trial; M, Male; F, Female.

↑ and ↓ indicate only the direction of changes reported in the original studies and are provided as a descriptive visual summary; they do not necessarily indicate a statistically significant between-group effect or superiority of dance over the comparator. ↔ indicates no statistically significant between-group difference. Detailed outcome instruments, time points, comparative results, and effect estimates, where reported, are presented in the narrative synthesis.

### 3.4 Intervention characteristics

Table 4 presents the key characteristics of the interventions included in the studies, including intervention type, duration, and frequency. The interventions comprised general dance (Wang et al., 2023; Ayari et al., 2023; Sánchez-Alcalá et al., 2025; Kattenstroth et al., 2013; Hola et al., 2024), ballroom dance (Ambegaonkar et al., 2022; Hirahara et al., 2025), square dance (Wu and Zhang, 2025; Zhao et al., 2021), dance movement therapy (Cofini et al., 2021; Ho et al., 2018), traditional Thai folk dance (Sanprakhon et al., 2025), digital exergaming dance (Tung et al., 2024), Latin dance (Wu and Zhang, 2025), and Zumba (Parial et al., 2023), as well as sports dance, line dance, tango, and therapeutic adapted dance. Some multi-arm studies included separate non-dance comparison arms, such as ukulele training, social conversation, martial arts, Tai Chi, Baduanjin, and orienteering (Ambegaonkar et al., 2022; Wu and Zhang, 2025; Hola et al., 2024; Zach et al., 2015). Other studies evaluated multicomponent interventions in which dance was combined with traditional cognitive treatment, cognitive stimulation, concurrent cognitive activities, or visual/multisensory stimulation (Cofini et al., 2021; Parial et al., 2023; Sanprakhon et al., 2025; Luo et al., 2026). The duration of the interventions ranged from 2 weeks (Aithal et al., 2019) to 6 months (Cofini et al., 2021; Tung et al., 2024; Kattenstroth et al., 2013). All studies reported the frequency of intervention delivery. Most interventions were conducted once or twice per week, while a smaller number were conducted three times per week. Intervention frequency generally ranged from one to three sessions per week. Session duration ranged from 30 to 90 min. Where reported, intervention intensity was generally moderate; however, intensity was not reported in several studies.

Among the 22 included studies, 18 were randomized controlled trials and four were non-randomized controlled studies. Regarding comparator conditions, the studies used health education, usual lifestyle or normal daily life, aerobic or other exercise, social conversation, traditional cognitive treatment, no-dance or no-intervention conditions, wait-list control, routine care, regular physical education, mindfulness meditation, and circuit physiotherapy. In addition, one study compared five groups: Tai Chi, Baduanjin, square dance, Latin dance, and a control group receiving health education (Wu and Zhang, 2025).

### 3.5 Outcome synthesis

#### 3.5.1 The effects of dance-based physical activity on cognitive function

Cognitive outcomes were examined across several domains, including global cognitive function, memory, attention, executive function and spatial perception. Most cognitive evidence remained derived from studies involving older adults, particularly those with mild cognitive impairment or cognitive decline, although two studies examined cognitive outcomes in children. Although several studies reported favorable changes in global cognitive function, the findings were not consistent across all populations or outcome measures. For example, Ho et al. (2018) reported no significant improvement in cognitive function among older adults with mild dementia. No cognitive outcomes were assessed in the studies involving adolescents or in the adult samples. The only study involving middle-aged adults did not assess cognitive outcomes. Therefore, the available evidence does not support consistent cognitive benefits across all age groups.

Ambegaonkar et al. (2022) assessed global cognitive function using the Montreal Cognitive Assessment in community-dwelling older adults who participated in ballroom dance, ukulele training, or social conversation programs. In the dance group, the MoCA score increased from 25.8 ± 2.5 at baseline to 27.3 ± 1.9 after the 10-week intervention and remained at 27.3 ± 2.0 at the one-month follow-up. However, cognitive scores also improved in the music and social conversation groups, and no significant difference was observed between groups (*p* = 0.60). Therefore, although cognitive performance improved over time, the study did not demonstrate that dance was superior to the other intervention conditions.

Additional studies involving older adults reported favorable but non-uniform cognitive findings. Sánchez-Alcalá et al. (2025) found that post-intervention scores favored dance-based aerobic training for global cognition measured using the MMSE (d = 0.96) and MoCA (d = 1.29), as well as verbal fluency (d = 1.04) and TMT-A performance (d = 0.64); however, the between-group effect for TMT-B was negligible (d = 0.05). Zhao et al. (2021) reported higher MoCA-P scores following square dance than health education both immediately after the intervention and at the three-month follow-up, although this was a non-randomized pilot study. Sanprakhon et al. (2025) reported favorable changes in subjective cognitive decline and cognitive functioning after the seven-week intervention and at the 12-week follow-up. However, traditional Thai folk dance was combined with cognitive stimulation, so the findings cannot be attributed specifically to dance. In Tung et al. (2024), the estimated between-group difference in MoCA change favored digital somatosensory dance, but this difference was no longer statistically significant after correction for multiple comparisons, and no significant between-group differences were observed for the assessed MoCA subdomains. Wang et al. (2023) reported a favorable effect on portrait memory following aerobic dance, whereas the other assessed cognitive outcomes did not show significant intervention effects. Wu and Zhang (2025) compared Tai Chi, Baduanjin, square dance, Latin dance, and health education. Cognitive improvement was reported for the square-dance and Tai Chi groups; therefore, among the eligible dance arms, the favorable cognitive finding applied to square dance rather than Latin dance, while results from the non-dance Tai Chi and Baduanjin arms were not interpreted as evidence for dance. Taken together, these studies support possible improvements in selected measures of global cognition, memory, verbal fluency, and executive function, but the findings varied across measures and intervention conditions and were not consistently observed across all dance interventions.

Cofini et al. (2021) conducted a randomized controlled trial (RCT) in children aged 9–11 years with specific learning disorders (SLD), using experimental dance therapy combined with traditional cognitive treatment. After the six-month intervention, the dance therapy group showed significantly higher adjusted scores than the control group for auditory recognition (26.29 ± 0.56 vs. 24.58 ± 0.52, *p* = 0.034) and visual recognition (25.31 ± 0.92 vs. 21.73 ± 0.94, *p* = 0.012). However, no significant between-group differences were observed for spatial recognition, digit span, Stroop performance, or planning ability. These findings suggest improvements in selected aspects of attention and recognition rather than consistent benefits across all cognitive domains.

Ayari et al. (2023) compared a 16-week dance intervention with an aerobic exercise program in 23 older adults with cognitive impairment. The dance group showed an increase in MMSE scores from 20.1 ± 3.5 to 23.3 ± 3.8, whereas scores in the aerobic exercise group remained largely unchanged (21.2 ± 3.5 to 21.1 ± 4.6). A significant group-by-time interaction was observed for global cognitive function, and the dance group showed a within-group improvement of 3.3 points (14%). Recall performance also improved in the dance group from 0.4 ± 0.7 to 1.4 ± 1.1, but not in the aerobic exercise group. The group-by-time interaction for recall was significant (*p* = 0.03, η^2^p = 0.20), with a within-group effect size of d = 0.95. However, no significant improvements were observed in the other MMSE cognitive domains.

Compared with the control group, a 12-week dual-task Zumba Gold dance program was associated with improvements in immediate and delayed recall in patients with MCI (Parial et al., 2023), whereas working memory did not change significantly. These findings suggest that improvements were observed in selected memory domains but were not consistent across all memory measures.

Evidence concerning attention and spatial perception was mixed across studies and measurement conditions. In Hola’s et al. (2024) Trail Making Test (TMT), after 12 weeks of dance training, the dance group improved by an average of 4.5 s and 18.4 s on the two TMT measures. In addition, Kattenstroth’s et al. (2013) study showed that after a 6-month dance intervention, the error rate of the intervention group on the Frankfurt Attention Inventory (FAIR) decreased significantly (3.52 ± 0.77 before the intervention vs. 2.63 ± 0.52 after the intervention, *p* = 0.043), while the performance index in the cognition/attention domain increased from 0.64 ± 0.02 to 0.72 ± 0.02 (*p* ≤ 0.001). Individuals with poorer baseline attention showed more pronounced improvements following the dance intervention. Furthermore, a 6-month dance therapy intervention targeting children aged 9–11 years with specific learning disorders (SLD) also showed that the treatment group achieved significantly more correct responses in auditory recognition and visual recognition tasks (Cofini et al., 2021). However, no significant between-group differences were observed in digit span, Stroop performance, or planning ability. The favorable attention-related findings reported by Hola et al. (2024) and Kattenstroth et al. (2013) should be interpreted cautiously because both studies were judged as having a high risk of bias.

Zach et al. (2015) examined attention and spatial perception in 123 children aged 4–5 years using a non-randomized controlled design. For centrally presented stimuli, significant changes over time were observed in attention, timing, impulsivity, and hyperactivity, but the absence of group-by-time interactions indicated that these changes were not specific to the dance group. Under distracting conditions, significant group-by-time interactions were reported for attention (*F* (1,68) = 4.53, *p* < 0.05, η^2^ = 0.062) and impulsivity (*F*(1,67) = 9.77, *p* < 0.01, η^2^ = 0.126). Omnibus group-by-time interactions across the dance, orienteering, and control groups were also reported for overall spatial perception and two spatial-position measures. However, no interaction was found for cube height, and no significant changes were observed for cube color or number. Because intact kindergarten classes were allocated to the study conditions, these findings provide limited evidence of improvements in selected attention and spatial-perception outcomes.

Overall, the available evidence suggests possible improvements in selected cognitive domains, particularly among older adults with cognitive impairment and in selected attention and spatial-perception measures in children. However, the findings were not consistent across all outcomes. The two studies involving children also differed substantially: Cofini et al. evaluated dance therapy combined with cognitive treatment, whereas Zach et al. (2015) used a non-randomized design with serious risk of bias. Differences in measurement instruments, intervention characteristics, and study-level risk of bias limit the strength of the conclusions.

#### 3.5.2 The impact of dance-based physical activity on mental health

Psychological outcomes included depressive symptoms, anxiety, psychological and parenting stress, mood disturbance, emotion recognition, interpersonal relationships, self-satisfaction, psychological well-being, subjective well-being, and quality of life. Findings varied across outcome domains, intervention conditions, and participant populations.

Depressive symptoms were examined mainly in older adults, particularly those with MCI or mild dementia. Zhao et al. (2021) reported a significant group-by-time interaction for GDS-30 scores following a square-dance intervention in older adults with MCI and depressive symptoms. At the end of the intervention, 24 of 31 participants in the intervention group changed from a mildly depressive to a non-depressive state; this number decreased to 16 participants at the three-month follow-up. Ho et al. (2018) similarly found that dance movement therapy produced small short-term reductions in depressive symptoms (d = 0.33), loneliness (d = 0.42), and negative mood (d = 0.30) compared with a waitlist control. The matched physical exercise condition did not show statistically significant effects compared with the waitlist control, although the psychological differences between the dance movement therapy and waitlist groups were no longer evident at the one-year follow-up.

Favorable findings were also reported by Hola et al. (2024), in which the GDS score in the dance group decreased from 2.3 ± 2.2 to 1.2 ± 1.3, and the adjusted change differed significantly from that in the inactive control group. In contrast, Ayari et al. (2023) observed reductions in GDS scores over time in both the dance and aerobic exercise groups, without a significant group-by-time interaction for depressive symptoms. Parial et al. (2023) also reported no significant improvement in depressive symptoms following the Zumba intervention. Among adults with self-reported depression, Pinniger found an overall between-group difference in depressive symptoms after the six-week intervention [*F*(2,59) = 6.00, *p* = 0.004, partial η^2^ = 0.17, 32]. Argentine tango was associated with lower post-intervention depression than the waitlist condition (*p* = 0.010, d = 0.50), but mindfulness meditation also differed from the waitlist condition (*p* = 0.025, d = 0.54), and the study did not demonstrate that tango was superior to the active meditation comparator. Moreover, only 66 of the 97 randomized participants were included in the analysis. Therefore, although selected studies reported reductions in depressive symptoms, the findings were not consistent and did not uniformly demonstrate a dance-specific advantage over active comparison interventions. Confidence in the favorable findings was further limited by the high risk of bias in Hola et al. (2024) and Pinniger et al. (2012), and the serious risk of bias in the non-randomized study by Zhao et al. (2021).

Anxiety outcomes were examined in two studies involving children and adolescents. Gao reported a group-by-time interaction for GAD-7 scores among adolescents exposed to academic stress (*F* = 68.32, *p* < 0.001, partial η^2^ = 0.58, 28). Compared with the control group, the ballroom-dance group had lower anxiety scores immediately after the 12-week intervention and at the four-week follow-up. Salivary cortisol showed a similar pattern, although it was a physiological rather than a psychological outcome. This study was judged as having some concerns regarding risk of bias. Luo et al. (2026) also identified group-by-time interactions for total social anxiety, fear of negative evaluation, and social avoidance and distress. However, post-intervention comparisons showed significantly lower anxiety than the control condition only for line dance combined with visual stimulation and for the multimodal line-dance condition; standard line dance alone was not shown to be superior to the control condition. Consequently, these findings cannot be attributed solely to line dance. Although a follow-up was described in the study protocol, follow-up results were not reported in the available outcome tables.

Findings for psychological and parenting stress were likewise mixed. In Pinniger et al. (2012), tango produced a moderate reduction in stress relative to the waitlist condition (*p* = 0.022, d = 0.45), whereas between-group effects for anxiety, self-esteem, and life satisfaction were not statistically significant. Zheng reported that scores across eight stress-response dimensions indicated less severe stress following an eight-week ballroom-dance intervention in university students and were significantly more favorable than those of the control group (Zheng and Ji, 2021). However, exact between-group estimates, confidence intervals, and effect sizes were not provided. In a small non-randomized study involving 11 mothers of children with autism spectrum disorder (ASD), Aithal et al. (2019) reported reductions in parental distress, parent–child dysfunctional interaction, and difficult-child scores following dance movement psychotherapy. Nevertheless, the groups differed substantially in baseline depression, and no valid comparative effect on depressive symptoms was established. Taken together, incomplete statistical reporting, the very small sample, baseline imbalance, and substantial risk-of-bias concerns limit the interpretation of these stress-related findings.

Findings for mood, emotional functioning, and self-related outcomes were also heterogeneous. Hirahara et al. (2025) found that, after a 10-week ballroom dance intervention, the overall level of emotional disturbance in the dance group decreased, with the POMS-2 Total Mood Disturbance (TMD) score dropping from 49.8 to 44.1, whereas the control group showed an upward trend. However, the study included only 15 participants per group and used non-random allocation. Among children with SLD, Cofini et al. (2021) found higher adjusted post-intervention scores for interpersonal relationships (*p* = 0.006) and emotion recognition (*p* = 0.016) in participants receiving dance therapy combined with traditional cognitive treatment compared with traditional cognitive treatment alone. Because dance therapy was delivered as an adjunct to cognitive treatment, these findings represent the effect of the combined intervention rather than stand-alone dance therapy.

Zheng reported significant interactions for personal ability and independence (*p* = 0.034), interpersonal relationships (*p* = 0.046), family emotion and interaction (*p* = 0.040), and overall self-satisfaction (*p* = 0.007). However, interactions were not significant for social identity and morality, family and economic background, or physical fitness and appearance. Similarly, Delextrat found interactions favoring Zumba for autonomy and purpose in life, but not for environmental mastery, personal growth, positive relationships, or self-acceptance. Thus, the findings supported changes in selected dimensions rather than a general improvement across all aspects of psychological well-being. Both studies were judged as having a high risk of bias.

Favorable findings for subjective well-being or quality of life were reported in several studies involving older adults. In Kattenstroth et al. (2013), 76% of participants in the dance group reported feeling better and 64% reported feeling more vital after the intervention. However, these percentages represented descriptive self-evaluations within the intervention group rather than formal between-group effect estimates. Favorable changes in subjective well-being or quality of life were also reported following Zumba, traditional Thai folk dance, and digital somatosensory dance interventions (Parial et al., 2023; Sanprakhon et al., 2025; Tung et al., 2024). In the feasibility study by Adey-Wakeling et al., participants receiving therapeutic dance reported better energy/fatigue than those receiving circuit physiotherapy at week 10, although this outcome also differed between groups at baseline. Emotional well-being favored therapeutic dance at week 10, and a greater proportion of participants reported no anxiety or depression on the EQ-5D-5L. These differences were no longer statistically significant at the four-week follow-up. As this was a small feasibility study not powered to establish effectiveness, these findings should be interpreted as preliminary.

Overall, dance-based interventions were associated with favorable changes in selected measures of depression, anxiety, stress, emotional functioning, self-satisfaction, and well-being. However, beneficial findings were not consistent across all outcome dimensions, were often absent against active comparators, and were not always maintained at follow-up. Interpretation is further constrained by small samples, combined or multimodal interventions, incomplete reporting of comparative estimates, and the high, serious, or critical risk of bias in several studies. The evidence therefore supports possible benefits for specific psychological outcomes rather than a consistent general effect of dance-based physical activity on mental health.

#### 3.5.3 Distribution of evidence and findings across age groups

This review included participants ranging from children to older adults. The 22 included studies involved 1,645 participants aged 4–94 years. The evidence comprised three studies involving children, one involving adolescents, four involving younger or middle-aged adults, one involving a broader adult age range, and 13 involving older adults. Because some adult samples crossed conventional age boundaries, these categories describe the populations reported by the individual studies rather than prespecified age-defined subgroups. Therefore, age-group findings were summarized descriptively rather than interpreted as direct comparisons of intervention effectiveness.

Among children, three studies examined cognitive or psychological outcomes. Zach et al. (2015) reported favorable changes in selected measures of attention and spatial perception among children aged 4–5 years. However, allocation was based on pre-existing kindergarten classes. Cofini et al. (2021) reported favorable findings for selected attention and concentration outcomes, including auditory and visual recognition, as well as interpersonal relationships and emotion recognition. However, no significant differences were observed for the assessed executive-function measures or for the body-self, school, and family dimensions of self-esteem. Because dance therapy was combined with traditional cognitive treatment, these findings cannot be attributed solely to dance therapy. Luo et al. (2026) reported reductions in social anxiety among children aged approximately 10 years, but post-intervention differences from the control group were identified only when line dance was combined with visual stimulation or multimodal sensory components; standard line dance alone was not shown to be superior to the control condition. Therefore, the children’s findings were outcome-specific and, in two studies, were limited by either combined interventions or non-random allocation.

Only Gao (Dongquan et al., 2026) specifically examined adolescents. Ballroom dance was associated with lower anxiety than the control condition immediately after the intervention and at the four-week follow-up. However, the study did not assess cognitive outcomes and was judged as having some concerns regarding risk of bias. Consequently, evidence for adolescents remains limited to one study and one principal psychological outcome.

Evidence involving younger and middle-aged adults was varied in terms of participant characteristics and outcome selection. Among university students, Zheng and Ji (2021) reported favorable changes in psychological stress and selected dimensions of self-satisfaction, although several self-satisfaction dimensions did not show significantly different changes over time between the two groups. Delextrat et al. (2016) reported improvements in autonomy and purpose in life among healthy young women, but not across the other assessed dimensions of psychological well-being. Aithal et al. (2019) reported reductions in parenting stress among caregivers aged 28–35 years, although the study included only 11 participants. Among adults aged 30–64 years, Hirahara et al. (2025) reported a favorable change in total mood disturbance following ballroom dance. Pinniger et al. (2012), whose sample covered a broader adult age range, found reductions in depression and stress compared with a waitlist control but did not establish superiority over mindfulness meditation. Overall, these findings were confined to selected outcomes and did not demonstrate a consistent pattern across adult populations. Furthermore, all five studies had substantial risk-of-bias concerns, limiting the strength of any age-specific conclusions.

The evidence base was largest for older adults, particularly those with MCI or other forms of cognitive impairment. Selected studies reported favorable changes in global cognition, memory, attention, verbal fluency, depressive symptoms, subjective well-being, and quality of life. However, the findings were not uniform. For example, Ho et al. (2018) found no significant immediate post-intervention improvement in overall cognitive function compared with the waitlist control. Ayari et al. (2023) found no dance-specific advantage over aerobic exercise for depressive symptoms, and the short-term emotional well-being differences reported by Adey-Wakeling et al. (2026) were no longer statistically significant at the four-week follow-up. The latter was a small feasibility study that was not powered to establish effectiveness. Furthermore, several favorable findings among older adults were derived from studies rated as having a high or serious risk of bias, which reduces confidence in the observed effects.

Overall, the current evidence does not support definitive comparisons of intervention effects across age groups. The greater number of studies involving older adults indicates that this population has been studied more extensively, rather than demonstrating that dance interventions are more effective in older adults. The number, outcome coverage, and methodological quality of studies remained uneven across age groups. In particular, adolescent evidence was limited to one anxiety study, while the adult studies examined heterogeneous populations and outcomes. These differences prevent reliable conclusions about whether the effects of dance-based physical activity vary according to age.

## 4 Discussion

### 4.1 Summary of main findings

The findings indicate that dance-based physical activity may benefit selected cognitive and psychological outcomes, but the results were not consistent across outcome measures or comparator conditions. Favorable cognitive findings were reported mainly in older adults with MCI or other forms of cognitive impairment, whereas evidence for younger age groups remained limited and heterogeneous. Given the methodological variation and study-level risk of bias, these findings should be interpreted cautiously and do not establish that dance-based physical activity is more effective in any particular age group.

### 4.2 The effect of dance-based physical activity on cognitive function

The included studies examined several domains of cognitive function, including global cognition, memory, executive function, attention, verbal fluency, and spatial perception. Although favorable findings were reported for some cognitive outcomes, the results were not consistent across all studies or cognitive domains. Most of the cognitive evidence was derived from studies involving older adults, particularly those with MCI or cognitive decline, while only two studies examined cognitive outcomes in children and none of the studies involving adolescents or the adult populations assessed cognition. There was also considerable heterogeneity in intervention dosage: intervention duration ranged from 7 weeks to 6 months, frequency ranged from once to three times per week, and individual sessions lasted between 30 and 90 min. These differences, together with variations in participant characteristics and outcome measures, limited direct comparison across studies and made it difficult to determine the optimal intervention dose.

A previous network meta-analysis involving interventions lasting three months or longer reported favorable cognitive outcomes (Huang et al., 2022). However, this observation should not be interpreted as evidence that longer interventions are necessarily more effective, because intervention duration, frequency, and intensity varied simultaneously across studies. For example, an intervention lasting 6 months but conducted only once per week, and another lasting 3 months but conducted three times per week, may involve a similar total number of sessions, but differ substantially in the distribution and cumulative dosage of training. As none of the included studies directly compared different intervention dosages or formally examined a dose–response relationship, the optimal duration and frequency of dance-based interventions remain unclear.

Findings for global cognition and related cognitive measures varied across measurement instruments and comparator conditions. Favorable effects were reported in some studies using usual-lifestyle or health-education controls (Sánchez-Alcalá et al., 2025; Zhao et al., 2021), whereas studies with active comparator arms did not consistently demonstrate a dance-specific advantage (Ambegaonkar et al., 2022; Wu and Zhang, 2025; Ho et al., 2018). Interpretation was further limited by the use of a multicomponent intervention (Sanprakhon et al., 2025) and by an estimated between-group effect that did not remain statistically significant after correction for multiple comparisons (Tung et al., 2024).

The cognitive findings also appeared to vary across different populations. Among the studies included in this review, favorable cognitive outcomes were reported more frequently in older adults with MCI or cognitive impairment than in cognitively healthy older adults. One possible explanation is that individuals with impaired baseline cognitive function may have greater potential for measurable improvement, whereas cognitively healthy older adults may have less room for change. This interpretation is broadly consistent with external evidence. A meta-analysis involving cognitively healthy older adults reported small-to-moderate effects of dance on global cognitive function (Jaldin et al., 2024), whereas a review of older adults with MCI reported effect sizes of 0.61 for MoCA and 0.37 for MMSE (Prates et al., 2025). However, these effect estimates were derived from separate reviews and cannot be directly compared. Differences in participant characteristics, intervention protocols, outcome measures, and study quality may also explain the observed variation. Therefore, baseline cognitive status may be a potential source of variation, but the current evidence is insufficient to establish it as a moderator or to conclude that dance interventions are more effective in people with MCI than in cognitively healthy older adults. Because Zumba Gold was combined with concurrent cognitive activities, the observed effects cannot be attributed to dance alone.

The effects of dance interventions on different cognitive domains are not uniform. For memory, Parial et al. (2023) reported improvements in immediate and delayed recall following a dual-task Zumba intervention, whereas working memory did not improve significantly. Ayari et al. (2023) also reported a favorable change in recall among older adults with cognitive impairment, but the remaining MMSE cognitive domains did not improve significantly. Wang et al. (2023) reported a favorable effect on portrait memory, whereas the other assessed cognitive outcomes did not show significant intervention effects. Thus, the available findings indicate possible benefits for selected recall and memory measures rather than memory function as a whole. One possible explanation is that learning and performing dance require participants to encode and recall movement sequences, which may provide repeated stimulation of selected memory processes. However, this mechanism was not directly examined in the included studies. As supporting evidence from outside the present review, Wu et al. (2025) found that a 3-month aerobic dance intervention improved logical memory scores in older adults with amnestic MCI and was associated with enhanced structural connectivity within the hippocampus-hub temporal network. This external finding provides a possible explanation for the observed memory-related outcomes but should not be interpreted as direct evidence from the studies included in this review.

Findings for attention and executive function were mixed. The review by Prates showed that dance interventions had significant effects on both TMT-A and TMT-B (Prates et al., 2025), while several studies included in the present review reported improvements in more narrowly defined attention or executive-function measures. Hola reported that after a 12-week dance intervention, older adults showed significantly shorter completion times on both TMT-A and TMT-B, indicating improved attention (Hola et al., 2024), and Kattenstroth et al. (2013) reported improvements in selected attention measures. Sánchez-Alcalá et al. (2025) found a favorable effect on TMT-A but a negligible between-group effect on TMT-B. In addition, Cofini et al. (2021) reported favorable results for auditory and visual recognition in children, but found no significant between-group differences in digit span, Stroop performance, or planning ability. Zach et al. (2015) reported favorable group-by-time interactions for selected attention, impulsivity, and spatial-perception measures under particular testing conditions, whereas several other measures showed no intervention-specific effects. Therefore, the current evidence suggests possible improvements in selected attention-related outcomes, rather than consistent improvements in executive function as a whole.

These findings should also be interpreted in relation to methodological quality. Several favorable cognitive findings were derived from studies judged to have a high or serious risk of bias, and some findings were based on combined interventions, specific subscales, or effects that were not maintained after adjustment for multiple comparisons. Differences in outcome instruments and statistical reporting further limited comparison of effect magnitude. Consequently, confidence in the consistency and magnitude of the observed cognitive effects remains limited.

External neuroimaging evidence suggests that dance and repetitive physical exercise may produce different patterns of brain adaptation. A study by Rehfeld et al. (2018) used magnetic resonance imaging and found that a six-month dance intervention was associated with larger volume increases in several brain regions, including the cingulate cortex, insula, corpus callosum, and sensorimotor areas. However, both intervention groups improved in attention and spatial memory, and no significant between-group differences were found for these cognitive outcomes. The structural findings may be related to the combined physical and cognitive demands of dance, including remembering movement sequences, following musical rhythm, adjusting spatial orientation, and coordinating movements with other participants. Nevertheless, neurobiological outcomes were not directly assessed in most studies included in this review. Therefore, these findings provide a plausible explanation for the observed cognitive outcomes but do not establish that dance is superior to other forms of physical or cognitive training.

### 4.3 The effect of dance-based physical activity on mental health

Dance-based physical activity was associated with favorable changes in selected psychological outcomes, including depressive symptoms, anxiety, stress, mood, and subjective well-being. However, these findings were not consistent across outcome domains or comparator conditions. This finding is consistent with a recent meta-analysis. In particular, the systematic review and meta-analysis by Prudente et al. (2024) reported a moderate overall reduction in depressive symptoms among older adults. However, the reduction was significant when dance was compared with no intervention, but not when it was compared with other active interventions. In addition, no significant effect was found in the subgroup of older adults with MCI, and the certainty of the evidence was rated as very low. A similar pattern was observed in the present review. Selected studies reported reductions in depressive symptoms compared with inactive or waitlist controls, whereas Parial et al. (2023) found no significant improvement and Ayari et al. (2023) found no dance-specific advantage over aerobic exercise. Pinniger et al. (2012) also reported lower depression following Argentine tango than following a waitlist condition, but did not establish superiority over mindfulness meditation. These findings suggest possible benefits for depressive symptoms, particularly in comparison with inactive conditions, but do not demonstrate a consistent advantage over other active interventions. Zhao et al. (2021) found that some improvements in depressive symptoms were maintained at the three-month follow-up despite a decrease in the frequency, duration, and intensity of dance participation. However, fewer participants were classified as non-depressive at follow-up than immediately after the intervention. Therefore, whether long-term and regular participation is necessary to maintain these psychological benefits remains unclear.

Findings for the other psychological outcomes were similarly outcome-specific. Favorable changes were reported for selected measures of anxiety, psychological and parenting stress, mood disturbance, self-satisfaction, psychological well-being, and quality of life. However, improvements were not observed across all measures. For example, the anxiety findings reported by Luo et al. (2026) were limited to line dance combined with visual or multimodal sensory components, while standard line dance alone was not shown to be superior to the control condition. In addition, some short-term psychological differences were not maintained at follow-up (Ho et al., 2018; Adey-Wakeling et al., 2026). Therefore, the available evidence supports possible benefits for particular psychological outcomes rather than a general improvement in mental health.

The possible mechanisms through which dance improves mental health may operate at both the physiological and psychological levels. Physiologically, dance may influence neurochemical pathways involving endorphins, dopamine (Klaperski-van der Wal et al., 2025), and serotonin (Lopez-Nieves and Jakobsche, 2022), thereby potentially eliciting pleasurable feelings and alleviating negative emotional states. In particular, Lopez-Nieves and Jakobsche (2022) summarized a study involving adolescents with mild depression in which dance movement therapy was associated with an increase in plasma serotonin and a decrease in plasma dopamine, together with reduced psychological symptoms. However, peripheral biomarker changes do not necessarily reflect changes in the central nervous system, and these mechanisms were not directly examined in most studies included in the present review. Psychologically, dance offers individuals a non-verbal channel for emotional expression, which may help enhance self-efficacy and social engagement.

The social nature of dance should not be overlooked. In the studies included in this review, most interventions were conducted in group dance formats. Such social interaction itself may provide cognitive stimulation and emotional support, and may thereby contribute to improvements in quality of life. A meta-analysis by Prudente suggested that when dance interventions were compared with other active interventions, they did not show a significant advantage in reducing depression (Prudente et al., 2024). This suggests that the observed psychological effects may reflect a combination of dance-specific elements and non-specific components such as physical activity, music, structured participation, and social interaction. Moreover, several favorable findings were derived from small studies, combined or multimodal interventions, or studies judged to have a high, serious, or critical risk of bias. These limitations reduce confidence in the consistency of the observed psychological effects.

### 4.4 Interpretation of findings across age groups

The studies included in this review covered a broad age range, spanning from children to the oldest-old, thereby providing a basis for a preliminary and descriptive consideration of age-related findings. The 22 included studies involved participants aged 4–94 years, but the evidence remained unevenly distributed: three studies involved children, one involved adolescents, four involved younger or middle-aged adults, one included a broader adult age range, and 13 involved older adults. Because these categories reflect the populations reported in the individual studies rather than prespecified age-defined subgroups, direct comparisons of intervention effects across age groups were not possible.

In children, the potential of dance-based physical activity to enhance selected cognitive and psychological outcomes deserves particular attention. Zach et al. (2015) reported favorable findings for selected measures of attention and spatial perception, whereas Cofini et al. (2021) reported favorable attention- and emotion-related outcomes but not consistent improvements in executive function or self-esteem. Luo et al. (2026) reported reductions in social anxiety only when line dance was combined with visual stimulation or multimodal sensory components; standard line dance alone was not shown to be superior to the control condition. Therefore, the findings in children were outcome-specific and were limited by non-random allocation in one study and the use of combined interventions in two studies. More broadly, a previous systematic review reported that dance has multiple health-promoting benefits for children and adolescents at both the physiological and psychological levels (Tao et al., 2022). External studies not included in the present review have also reported favorable findings for selected aspects of executive function and working memory (He et al., 2025; Intawachirarat et al., 2025; Rudd et al., 2021). However, these external findings cannot compensate for the small and methodologically heterogeneous body of evidence included in the present review.

Further external evidence provides possible context for these findings. In a randomized repeated-measures study involving 38 children aged 7–12 years, He et al. (2025) found that a digital dance intervention accompanied by rhythmic music led to significant improvements in both inhibitory control and cognitive flexibility, suggesting that the combination of rhythmic auditory stimulation and motor training may exert positive effects on children’s executive function. In addition, Intawachirarat et al. (2025) reported that, among children with obesity aged 9–12 years, moderate-intensity contemporary dance training was associated with improved working memory within the intervention group and increased brain-derived neurotrophic factor (BDNF) levels. The concurrent changes in working memory and BDNF suggest a possible neurobiological explanation, although the study did not establish that changes in BDNF mediated the cognitive improvement. However, whether other activities that likewise require multitasking, such as musical instrument training or competitive sports, can produce similar effects in children remains to be further investigated.

Evidence for adolescents was more limited. Only Gao (Dongquan et al., 2026) specifically examined this age group and reported lower anxiety following ballroom dance than in the control condition immediately after the intervention and at the four-week follow-up. However, this study assessed one principal psychological outcome and did not examine cognitive function. Consequently, no conclusions can be drawn regarding broader cognitive or psychological effects in adolescents.

The studies involving younger and middle-aged adults examined heterogeneous populations and almost exclusively psychological outcomes (Zheng and Ji, 2021; Pinniger et al., 2012; Delextrat et al., 2016; Hirahara et al., 2025; Aithal et al., 2019). Favorable findings were reported for selected measures of stress, mood disturbance, self-satisfaction, parenting stress, and psychological well-being, but improvements were not observed across all measured dimensions. Moreover, the evidence included small samples and studies judged to have a high, serious, or critical risk of bias. Because no cognitive outcomes were assessed and the psychological measures differed substantially across studies, the available evidence does not support general conclusions regarding the effects of dance-based physical activity in younger or middle-aged adults. In recent years, studies of dance interventions targeting middle-aged populations have gradually increased. Lu et al. (2024) included 16 studies in a systematic review of middle-aged and older adults and found that dance interventions were associated with favorable findings for physical function, postural control, and quality of life, as well as higher adherence than some comparison interventions. However, the findings were not reported separately for middle-aged adults. Peng et al. (2024) similarly reviewed dance therapy in middle-aged and older adults with arterial hypertension and reported favorable effects on blood pressure; however, the findings were not reported separately for middle-aged adults and therefore do not provide age-specific evidence for cognitive or psychological outcomes in this population. Therefore, this external review supports the possible value of dance within a broader middle-aged and older population but does not provide age-specific evidence for middle-aged adults. Future research should separately examine and report cognitive and psychological outcomes in this population.

In older adults, particularly those with MCI, dance-based physical activity interventions more frequently produced favorable cognitive findings, although the results were not uniform and this pattern may partly reflect the substantially larger number of studies involving this age group. Several studies included in this review reported improvements in cognitive function among older adults with MCI or other forms of cognitive impairment following dance interventions (Parial et al., 2023; Sánchez-Alcalá et al., 2025; Sanprakhon et al., 2025; Zhao et al., 2021). However, differences in study design, outcome measures, comparator conditions and risk of bias limit confidence in the consistency of these findings. This finding is broadly consistent with the results of a recently published systematic review. Tao et al. (2023) conducted a systematic review and meta-analysis of 29 studies on dance interventions (including 13 RCTs with a total of 1,708 participants) and reported significant pooled improvements in global cognitive function, memory, and balance in individuals with MCI, Alzheimer’s disease, and dementia, as well as reductions in depressive symptoms. Another meta-analysis of eight RCTs also reported favorable effects of dance interventions on global cognitive function (Prudente et al., 2024). These external findings support the possible cognitive value of dance for older adults with cognitive impairment, but they do not establish that dance is consistently superior to other active interventions.

The apparently more favorable pattern among older adults with cognitive impairment may partly reflect greater potential for measurable improvement at baseline. External neuroimaging studies have also reported changes in brain connectivity and regional brain volume following dance interventions (Wu et al., 2025; Rehfeld et al., 2018), providing possible explanations for some of the observed cognitive findings. However, the available evidence does not establish whether these changes represent restoration of impaired neural function or compensation through alternative neural processes. This interpretation therefore remains preliminary.

Overall, the concentration of favorable findings among older adults should not be interpreted as evidence that dance-based physical activity is more effective in older than in younger populations. It may instead reflect the greater number of studies involving older adults, differences in baseline cognitive or clinical status, and variation in the outcomes, interventions, and comparator conditions used across age groups. The available evidence therefore permits only a descriptive interpretation of age-related patterns and does not support definitive conclusions about whether the effects of dance-based physical activity vary according to age.

## 5 Limitations and heterogeneity

Although favorable effects of dance interventions were reported, this review identified substantial heterogeneity across studies. In terms of intervention type, the included dance interventions ranged from ballroom dance, square dance, and Zumba to traditional folk dance, dance movement therapy, digital dance, therapeutic dance, and line-dance programmes with additional sensory components. Different types of dance vary in intensity, cognitive load, and the degree of social interaction. In terms of intervention frequency, the frequency ranged from once to three times per week, and the duration varied from 2 weeks to 6 months, which may influence the stability and durability of the intervention effects. Intervention intensity was also not reported in several studies, further limiting comparison of intervention dosage. Regarding control group design, some studies used usual lifestyle, routine care, or health education as controls, whereas others used other forms of physical activity (such as aerobic exercise), physiotherapy, mindfulness meditation, or socially engaging activities, making direct comparisons difficult.

Existing studies also have several methodological limitations. Prudente reported that research in this field is characterized by a moderate risk of bias and very low certainty of evidence (Prudente et al., 2024). Among the studies included in this review, most had small sample sizes (11–204 participants) and were predominantly female, which may have introduced bias and limited the generalizability of the findings to other populations. Methodological quality was also a concern: most randomized studies were judged to have some concerns or a high risk of bias, while the four non-randomized controlled studies were judged to have a serious or critical risk of bias. In addition, the study populations were still mainly concentrated in older adults, with relatively limited research on children, adolescents, younger or middle-aged adults. Although the updated review included three studies involving children, one involving adolescents, four involving younger or middle-aged adults, and one involving a broader adult age range, 13 of the 22 studies involved older adults. The number, outcome coverage, and methodological quality of studies therefore remained uneven across age groups. This imbalance reflects a gap in the literature and limits the ability to draw definitive conclusions regarding the effects of dance interventions across different age groups. Future research should include more children, adolescents, and middle-aged adults. Furthermore, long-term follow-up data were limited, preventing firm conclusions about the sustainability of intervention effects. Neuroimaging studies also remain relatively scarce, and current understanding of the neural mechanisms underlying dance interventions is still largely inferred from behavioral indicators.

The interpretation of the findings was further limited by differences in intervention composition and comparator conditions. Some interventions consisted of dance alone, whereas others combined dance with cognitive treatment or other components, making it difficult to attribute observed effects specifically to dance. In addition, studies using passive controls may have been more likely to detect favorable effects than those using active exercise or socially engaging controls. Outcome measures and statistical reporting also differed substantially, preventing reliable comparison of effect magnitude across studies. Some studies reported between-group or group-by-time effects, whereas others primarily reported within-group changes or did not provide effect estimates and confidence intervals. Because a meta-analysis and formal certainty-of-evidence assessment were not conducted, the conclusions should be regarded as descriptive and preliminary. In addition, 59 reports could not be retrieved despite searches through the university library’s subscribed databases and Google Scholar and DOI-based open-access checks in Unpaywall; therefore, some potentially eligible evidence may have been missed. In addition, only English-language publications were eligible, which may have introduced language bias and resulted in the exclusion of relevant studies published in other languages.

## 6 Clinical implications and future directions

Based on the current evidence, dance-based physical activity may be considered a potentially useful adjunctive non-pharmacological approach, particularly in older populations for whom the available evidence is most extensive, rather than an established intervention across different age groups. If used in practice, the dance form and programme should be adapted to the age, health status, physical ability, preferences, and safety requirements of the target population. For children and adolescents, the current evidence is insufficient to recommend a particular dance form for improving executive function or psychological health. For older adults, especially those with mild cognitive impairment (MCI), dance-based activities may support cognitive and psychological health; however, the current evidence is insufficient to recommend a particular dance form, intensity, or dosage. Evidence involving children, adolescents, younger and middle-aged adults also remains too limited and heterogeneous to support age-specific recommendations.

Future research should focus on several directions. First, large-sample, preregistered randomized controlled trials are needed, particularly in underrepresented populations such as children, adolescents, and younger and middle-aged adults. These studies should use appropriate active comparison groups, standardized outcome measures, and longer follow-up periods to determine the durability of the effects. Direct comparisons of different dance forms and intervention dosages are also required to identify the optimal frequency, intensity, and duration. Second, future studies could, where appropriate, combine neuroimaging techniques (such as fMRI and DTI) with biomarkers (such as BDNF and IGF-1) to further clarify the neural mechanisms underlying dance interventions. Third, the feasibility of remotely supervised or digital dance interventions should be explored in order to improve accessibility and adherence.

## 7 Conclusion

This systematic review synthesized 22 studies involving 1,645 participants aged 4–94 years. Dance-based physical activity may have favorable effects on selected cognitive outcomes, including global cognition, memory, attention, verbal fluency, and spatial perception, as well as selected psychological outcomes such as depression, anxiety, stress, mood, and well-being. However, these findings were inconsistent across outcome measures and comparator conditions, and several favorable results arose from studies with substantial risk-of-bias concerns. The evidence was concentrated in older adults, particularly those with cognitive impairment, and does not support conclusions that dance is more effective in one age group than another. The evidence base is limited by methodological heterogeneity, small sample sizes, risk of bias, uneven age-group representation, combined or multimodal interventions, and limited long-term follow-up data. Future research should prioritize standardized intervention protocols, appropriate active comparators, adequately powered trials in underrepresented age groups, comparisons of different dance types, and studies incorporating neuroimaging techniques. From a public health perspective, dance represents an enjoyable and socially engaging activity that may serve as an adjunctive non-pharmacological strategy for supporting selected aspects of cognitive and mental well-being, particularly among older adults. However, the current evidence is insufficient to support definitive conclusions about its effectiveness across the lifespan.

## Data Availability

The original contributions presented in the study are included in the article/supplementary material, further inquiries can be directed to the corresponding author.
